# Mobile Health Apps for Individuals Who Are Blind or Have Low Vision: Systematic Review of Evidence, Accessibility, and App Quality

**DOI:** 10.2196/85809

**Published:** 2026-11-12

**Authors:** Carlos Mora, Ester Solé, Pere Llorens-Vernet, Jordi Miró

**Affiliations:** 1 Research Center for Behavior Assessment (CRAMC), Department of Psychology, Universitat Rovira i Virgili, Carretera de Valls s/n, Tarragona, Catalonia, 43007, Spain, 34 649298921; 2 Unit for the Study and Treatment of Pain–ALGOS, Chair in Pediatric Pain, Tarragona, Catalonia, Spain; 3 Institut d’Investigació Sanitària Pere Virgili, Tarragona, Catalonia, Spain

**Keywords:** blindness, visually impaired persons, mobile apps, mHealth, telemedicine, mental health, self-management, accessibility

## Abstract

**Background:**

Individuals who are blind or have low vision experience disproportionate burdens in both physical and psychological functioning, and mobile health apps may help address some of these needs. However, their availability, accessibility, and empirical support for this population remain unclear.

**Objective:**

This systematic review aimed to identify mobile health apps relevant to supporting physical and psychological functioning in individuals who are blind or have low vision, summarize the empirical evidence available for these apps, and examine their accessibility and quality.

**Methods:**

We used a multicomponent approach that combined (1) identification of apps through organizational recommendations; (2) a systematic search of the scientific literature, conducted and reported in accordance with PRISMA (Preferred Reporting Items for Systematic Reviews and Meta-Analyses) guidelines; and (3) an exploratory accessibility and app quality appraisal using the Web Content Accessibility Guidelines 2.1 and the Mobile App Development and Assessment Guide.

**Results:**

A total of 19 unique mobile health apps were identified after combining and deduplicating apps identified through organizational recommendations and scientific literature. Fifteen apps were identified through organizational recommendations, and 14 empirical studies evaluating 11 apps were included in the qualitative synthesis. Only a subset of these studies specifically involved blind or low-vision participants; several evaluated apps in broader clinical or general populations where visual impairment status was not reported. The identified apps focused primarily on physical functioning, particularly medication management, glucose monitoring, pharmaceutical information access, and health care access. No apps primarily designed to support psychological functioning in individuals who are blind or have low vision were identified. Of 14 studies, 6 (43%) were rated as being of good methodological quality. The exploratory user-informed accessibility appraisal showed variability across apps: MyPills received the most favorable accessibility profile, with “High” ratings across all 4 Web Content Accessibility Guidelines principles, whereas several organizationally recommended apps received low ratings across most principles. Mobile App Development and Assessment Guide scores ranged from 8/39 to 34/39, with Medisafe scoring highest.

**Conclusions:**

The current landscape of mobile health apps for individuals who are blind or have low vision remains limited, uneven in accessibility and quality, and supported by a limited and heterogeneous evidence base. The absence of identified apps targeting psychological functioning represents a particularly important gap. Improving the development and implementation of mobile health apps for individuals who are blind or have low vision will require more than increasing the number of available apps; it will require apps that are accessible from the outset, rigorously evaluated, and developed with the people they are intended to serve.

## Introduction

In 2019, the World Health Organization estimated that at least 2.2 billion people worldwide are blind or have low vision [1], and the number of individuals living with blindness is projected to reach 115 million by 2050. Visual impairment is therefore a major global public health issue with substantial implications for health, functioning, and quality of life [2].

Research consistently shows that individuals who are blind or have low vision report poorer physical and mental health outcomes than their sighted peers [3-6]. For example, a recent review [7] concluded that chronic pain, particularly neuropathic ocular pain and back pain, appears to be common in this population. Visual impairment is also a well-established risk factor for falls and fractures [8,9], which may in turn contribute to loss of independence, permanent disability, and increased mortality, especially in older adults [10]. Mental health outcomes are similarly affected. Adults with visual impairments experience higher rates of depression [11] and anxiety [4] than those with normal vision, and greater severity of visual impairment has also been associated with increased risk of suicidal thoughts and behaviors [12,13].

Despite the elevated risks, access to health care remains limited for individuals with disabilities, including those with visual impairments [14]. Previous research has documented persistent disparities in preventive care, health care usage, and access to appropriate treatment compared with sighted individuals [2,15,16]. For example, although older adults with visual impairments present higher rates of clinically significant depression and anxiety [17], many never receive appropriate treatment [18,19]. Even in settings where accessibility is legally mandated, implementation is inconsistent [20]. Access to effective treatment is further restricted by the limited accessibility of many medical technologies. For example, insulin pumps and other diabetes-related devices often lack basic accessibility features such as voice guidance and accessible instructions [21], despite the relevance of diabetes in this population.

Mobile health apps (ie, software designed for smartphones, tablets, and other portable devices [22]) have emerged as promising tools to support the management of chronic noncommunicable diseases, such as chronic pain [23,24], diabetes [25], hypertension [26], depression [27], and anxiety [28]. For individuals who are blind or have low vision, mobile apps can promote autonomy, enhance communication with health care professionals and peers, and improve personalized care and overall quality of life [29-33]. However, many commercially available apps are developed primarily for commercial rather than clinical purposes and lack rigorous empirical support [34]. Limited involvement of key stakeholders, particularly end users [35], may further contribute to mistrust, privacy concerns, and the dissemination of inaccurate or even potentially harmful information [36,37]. Some reviews and empirical studies have begun to describe the broader landscape of mobile app usability for visually impaired users. For example, Al-Razgan and colleagues [29] conducted a systematic literature review on the usability of mobile apps for people with visual impairments, identifying key themes such as accessibility, assistive technologies, navigation, and interaction design. Their review provides an important overview of the general usability challenges faced by visually impaired users in mobile environments. However, mobile health apps, particularly those intended to support the physical and psychological functioning of individuals who are blind or have low vision, received far less systematic attention.

To our knowledge, no systematic review has specifically examined mobile health apps relevant to individuals who are blind or have low vision with a focus on their potential role in supporting physical and psychological functioning, the empirical evidence supporting their use, and the extent to which such apps meet current accessibility and quality standards. Conducting such a review has important implications for both research and clinical practice: it can help identify promising tools, clarify important gaps in the evidence base, and inform the development and recommendation of apps that are both effective and accessible for this population.

Accordingly, this systematic review addressed three research questions: (1) Which mobile health apps relevant to individuals who are blind or have low vision can be identified through organizational recommendations and the scientific literature? (2) What empirical evidence is available regarding the usability, accessibility, and health-related outcomes of these apps? (3) What do the exploratory user-informed accessibility appraisal and app-quality assessment suggest about the accessibility and quality of the identified apps?

## Methods

### Procedure

#### Overview

This study followed a multicomponent review design intended to address complementary dimensions of the same research problem. First, we identified mobile health apps recommended by organizations serving individuals who are blind or have low vision in order to characterize apps currently promoted within this community. Second, we conducted a systematic review of the scientific literature to identify and examine empirical studies evaluating mobile health apps relevant to this population. Apps identified through organizational recommendations and the scientific literature were then combined and deduplicated by app name, developer, intended function, and platform availability to determine the final number of unique apps included in the review. This review followed the PRISMA (Preferred Reporting Items for Systematic Reviews and Meta-Analyses) guidelines [38] for systematic reviews and meta-analyses and was conducted in accordance with the preregistered protocol in the PROSPERO [39] database (CRD42023448243). No substantive deviations from the protocol occurred; any minor refinements were limited to improvements in reporting clarity and did not affect the scope, eligibility criteria, or analytical approach. All studies were analyzed using a qualitative synthesis approach. Two independent reviewers (CM and JM) assessed study eligibility using a 2-step process that involved initial screening of titles and abstracts followed by full-text evaluation. Discrepancies were resolved through discussion, and when necessary, a third reviewer was consulted to reach consensus. Third, we carried out an exploratory, user-informed accessibility appraisal of apps that were available for direct review. This final component was not designed as a stand-alone primary study or as a formal usability trial intended for statistical generalization but rather as a complementary appraisal to provide practical insight into how the identified apps could be accessed and experienced by potential users. Accordingly, the accessibility appraisal was intended to contextualize the evidence synthesis by bridging the gap between published descriptions of apps and their real-world accessibility in use. As this review was based exclusively on previously published literature and did not involve the collection of primary data or direct participation of human subjects, approval from an Institutional Review Board (IRB) or Research Ethics Committee was not required.

#### Identification of Apps Recommended by Organizations Serving Individuals With Visual Impairments

To identify mobile health apps currently recommended to individuals who are blind or have low vision, we conducted a targeted review of websites and digital resources from organizations serving this population. These organizations often act as key sources of information and dissemination channels for assistive technologies and digital tools within the visually impaired community. Organizations from different geographical regions were considered, including international, national, and regional organizations that provide resources and support for individuals with visual impairments. Examples include the American Foundation for the Blind, the Royal National Institute of Blind People, the World Blind Union, the Spanish National Organization for the Blind (ONCE), and Vision Australia. The websites of these organizations were systematically examined to identify mobile health apps intended to support physical and psychological functioning in individuals who are blind or have low vision and those that were recommended or listed as potentially useful for this population. When relevant information was available, we recorded basic descriptive characteristics of each app, including the app name, platform availability (ie, iOS or Android), intended purpose, and any description provided by the recommending organization.

#### Systematic Search of the Scientific Literature on Mobile Health Apps Intended to Support Physical and Psychological Functioning

To identify empirical studies on mobile health apps intended to support the physical and psychological functioning of individuals who are blind or have low vision, we conducted a systematic search of the scientific literature. The search was performed in 3 major databases: ScienceDirect, PubMed or MEDLINE, and other National Center for Biotechnology Information (NCBI) resources. The search strategy combined terms related to mobile apps and visual impairment. Specifically, the following keywords were used: (“app*” OR “mobile” OR “smartphone”) AND (“visually impaired” OR “visual disability” OR “blind*” OR “low vision” OR “ocular*” OR “eye*”), using asterisks as wild cards to capture variations of each term. A supplementary app-name search was also conducted for apps identified through organizations serving individuals with visual impairments, combined with the same keyword strategy. The initial search was conducted in ScienceDirect, PubMed or MEDLINE, and other NCBI resources and covered records published through December 2025. In April 2026, the search was updated and extended across all 6 databases included in the final review: ScienceDirect, PubMed or MEDLINE, PubMed Central, Scopus, Web of Science, and the ACM Digital Library. All 6 databases were searched from inception through April 2026. Thus, the April 2026 update both extended the final coverage date of the databases included in the initial search and broadened database coverage by adding Scopus, Web of Science, and the ACM Digital Library. The updated search strategy was developed through an iterative refinement process. Initial exploratory searches using broad terms yielded a high proportion of irrelevant records due to the ambiguity of certain keywords. Therefore, search terms were progressively refined by prioritizing more specific descriptors related to mobile apps and visual impairment and by combining these with methodological terms (eg, “evaluation” and “empirical study”) where appropriate. This approach aimed to balance sensitivity and specificity, ensuring adequate coverage while reducing noise. The search was restricted to peer-reviewed journal papers. Gray literature and conference proceedings were not included as primary evidence sources, as the review focused on empirical studies with formal evaluation components to ensure methodological consistency and comparability across studies. The full database-specific search strategies used in the updated search, including complete search strings, Boolean operators, filters, and records retrieved per source, are provided in Multimedia Appendix 1. The supplementary app-name search was intended to identify empirical studies on apps already recommended in practice, while the broader database search aimed to capture additional relevant apps not identified through organizational sources.

To maximize comprehensiveness, we also manually screened the reference lists of eligible papers for additional studies. Studies were included if they met the following criteria: (1) evaluated a mobile app intended as relevant to individuals who are blind or have low vision, because it was designed for this population, recommended by organizations serving this population, or identified in the scientific literature as potentially relevant; (2) reported empirical findings related to the app; (3) were published in a peer-reviewed journal; (4) were available in full text; and (5) were written in English or Spanish. Studies were eligible even when the empirical evaluation was conducted in a broader clinical or general population, provided that the app itself had been identified as relevant to individuals who are blind or have low vision. Data extraction was performed using a structured template developed for this review. Extracted variables included study characteristics (authors, year, country, and design), participant characteristics, app name and purpose, health domain addressed, key accessibility and usability findings, and the main outcomes reported. Study designs were categorized into broader groups (eg, observational and experimental) to facilitate consistent reporting and comparison across heterogeneous studies. The data extraction form used in this review, including all extracted variables, is provided in Multimedia Appendix 2.

#### Assessment of Methodological Quality and Risk of Bias

Methodological quality and risk of bias were assessed by 2 reviewers (CM and JM) using the version of the National Institutes of Health (NIH) Quality Assessment Tool appropriate to each study design. Discrepancies were discussed and resolved by consensus following guidance from the Cochrane Handbook for Systematic Reviews of Interventions. The final ratings for all included studies were based on this consensus process.

For each study, the NIH criteria were rated as “yes,” “no,” “cannot determine,” or “not applicable,” as appropriate. “Cannot determine” was used when insufficient information was available to judge an item, and “not applicable” when a criterion was not relevant to the study design. To obtain an overall indicator of methodological rigor, “yes” responses were scored as 1 point, whereas all other responses (“no,” “not reported,” “cannot determine,” or “not applicable”) were scored as 0, and item scores were summed across criteria. Consistent with approaches used in previous systematic reviews [40-42], total scores were categorized as poor (0‐6), fair (7-10), or good (11-14). For ease of interpretation, the resulting ratings are presented both at the item level and as an overall summary classification.

#### Exploratory Accessibility and Functionality Appraisal of the Identified Mobile Apps

This appraisal was conceived as an exploratory complement to the review. Consequently, the resulting ratings should be interpreted as illustrative observations derived from participant experiences and consensus discussions rather than as a formal usability study or as independently generalizable evidence. The accessibility and functionality of the identified apps were appraised using the Web Content Accessibility Guidelines (WCAG 2.1, 2018) [43] framework focusing on the 4 POUR principles: (1) Perceivable (ie, information and user interface elements must be easily perceivable by users), (2) Operable (ie, all elements should be fully interactive, free of distracting animations, or gestures that require the use of multiple fingers), (3) Understandable (ie, information and interface functions should be clear and easy to understand), and (4) Robust (ie, apps should be compatible with different devices and operating systems, including features such as adjustable font sizes). In practical terms, the accessibility appraisal examined whether relevant app information and interface elements were perceivable (eg, accessible labels, readable content, and discernible feedback), whether navigation and controls were operable (eg, keyboard or screen-reader accessibility, predictable navigation, and actionable controls), whether content and interaction flows were understandable (eg, clarity of instructions, consistency, and comprehensibility of interface elements), and whether the app appeared robust in its compatibility with assistive technologies and platform accessibility services. Although WCAG was originally developed for web content, its principles have also been applied and adapted to mobile and nonweb software environments in World Wide Web Consortium (W3C) guidance and prior accessibility research [43,44]. Because some WCAG wording is web-oriented, criteria were interpreted in relation to mobile app environments following established W3C guidance for nonweb Information and Communication Technologies and mobile apps. Thus, references to web pages were considered at the level of app screens or views, and accessibility judgments were made in relation to native app interactions and compatibility with mobile assistive technologies. Following this approach, we applied WCAG 2.1 to native mobile apps to assess barriers and facilitators relevant to individuals who are blind or have low vision, particularly those related to screen reader compatibility, interface clarity, navigation, and interaction with core app functions.

To assess the accessibility and functionality of the identified apps, we conducted a user-based appraisal guided by the WCAG 2.1 framework. Four adult volunteers with visual impairments participated in the evaluation (2 blind and 2 with reduced visual acuity). Participants were recruited through convenience sampling based on accessibility and prior experience using mobile assistive technologies. No formal inclusion or exclusion criteria were applied beyond having lived experience of visual impairment and familiarity with smartphone use. Participants were instructed to download, install, and test the core functionalities of each app (eg, setting medication reminders and navigating menus) using the native accessibility features available on their devices. Following individual testing, participants discussed their experiences in a group session and appraised each app in terms of ease of use, clarity of information, compatibility with screen readers or other assistive technologies, and overall performance across the 4 WCAG principles. Based on this process, each app received an overall rating of low, moderate, or high for each WCAG principle. When disagreement occurred, the most stringent rating was retained in order to prioritize barriers encountered by blind users. These ratings therefore reflect a consensus-based synthesis of the participants’ practical experience with the apps rather than a formal psychometric assessment, standardized accessibility measurement, or clinical evaluation. No interrater reliability, test-retest reliability, or formal validation procedure was conducted for this exploratory component. A detailed operationalization of WCAG 2.1 principles, including examples, is provided in Multimedia Appendix 3. In addition, we reviewed scientific literature related to the accessibility and functionality of the identified apps to complement the user-based appraisal. Given the small and diagnostically homogeneous sample, this component was intended to enrich the review with real-world end user perspectives rather than to generate generalizable estimates of usability.

#### App Quality Appraisal Using the Mobile App Development and Assessment Guide

Each identified app was also appraised using the Mobile App Development and Assessment Guide (MAG) [35]. The MAG is a structured framework developed to support the assessment of mobile health apps across multiple domains relevant to quality, including usability, privacy, security, appropriateness, safety, and technology performance. The guide includes 39 items organized into seven categories: (1) Usability, (2) Privacy Policy Information, (3) Privacy Features, (4) Security, (5) Appropriateness and Suitability, (6) Safety, and (7) Technology Performance [34]. Two independent researchers (CM and PLl-V) reviewed each app against the 39 MAG criteria. Each item was rated as “yes” when the criterion was met, “no” when it was not met, and “not applicable” when the criterion could not reasonably be assessed for a given app. Agreement between reviewers was approximately 90% before consensus discussions. Disagreements were generally minor and primarily reflected different interpretations of individual MAG criteria. These discrepancies were resolved through discussion and consensus review before final scores were assigned. The full list of MAG criteria used in the evaluation is presented in Table 1. In addition to the item-level appraisal, an overall summary score was calculated for each app based on the number of MAG criteria met, in order to facilitate comparison across apps. Because the MAG does not provide predefined cutoff points for interpreting total scores, descriptive score bands were created for this review. Apps meeting approximately two-thirds or more of the MAG criteria were classified as higher app quality (≥27/39), apps meeting approximately one-half to less than two-thirds of the criteria were classified as moderate app quality (20-26/39), and apps meeting fewer than one-half of the criteria were classified as lower app quality (≤19/39). These categories were used only to aid interpretation of the descriptive MAG scores and should not be interpreted as validated MAG thresholds.

**Table 1.** Mobile App Development and Assessment Guide (MAG) criteria used to appraise the quality of mobile health apps for individuals who are blind or have low vision.

Item	Statement	MAG category
1	The app was tested by potential users before being made available to the public.	Usability
2	It is easy to use.	Usability
3	The app features are adapted to the purposes of the app.	Usability
4	Functionality is adjusted to the targeted user.	Usability
5	The app is designed to be accessible for people with physical impairments.	Usability
6	The app uses lay language that can be easily understood by the targeted user.	Usability
7	The user can easily access all resources and features.	Usability
8	The app provides information about the terms and conditions for the use of the app.	Privacy Policy Information
9	The app provides information about privacy policies.	Privacy Policy Information
10	The app provides information about commercial agreements with third parties or the absence of them.	Privacy Policy Information
11	The app informs users if their data must be transferred to third parties for the app to operate.	Privacy Policy Information
12	The app indicates a recommended user age based on its content.	Privacy Policy Information
13	The app includes a simple and understandable version of the terms and conditions of use, and privacy policies.	Privacy Policy Information
14	The app gives a warning when an update influences sensitive data.	Privacy Features
15	The app provides the option to only collect user data that are essential for the app to operate.	Privacy Features
16	The app guarantees users' rights on stored personal data.	Privacy Features
17	The app provides the user with the option to refuse the transfer of data to third parties.	Privacy Features
18	The app requires the user to give her or his consent to the terms and conditions of use and privacy policies.	Privacy Features
19	The app notifies the user when other resources on the mobile device, such as her or his accounts or social network profiles, are accessed.	Privacy Features
20	The app guarantees the user’s privacy.	Privacy Features
21	The app offers to delete the user’s data at any time.	Privacy Features
22	The app informs the user about the security measures in place.	Security
23	The app includes password management mechanisms.	Security
24	The authorization and authentication mechanisms work correctly.	Security
25	The app validates the user’s identity to access confidential data.	Security
26	The app informs the user when it detects and identifies cybersecurity vulnerabilities, possible threats, and the risk of being exploited that may affect the data.	Security
27	The app informs the user that when a third-party component is updated, the updated version is inspected, and the risks are evaluated.	Security
28	The benefits and advantages of using the app are explained.	Appropriateness and Suitability
29	Experts have participated in the development of the app.	Appropriateness and Suitability
30	The app informs the user that it is based on scientific findings.	Appropriateness and Suitability
31	The app informs the user that it is developed in accordance with ethical principles and values.	Appropriateness and Suitability
32	The possible health risks for the user are identified.	Safety
33	The app complies with regulatory standards as a medical device.	Safety
34	The user is warned when adverse events are identified.	Safety
35	The app recommends that users always consult a specialist if they have questions, or it warns users that the app is not meant to replace the services provided by a health professional.	Safety
36	The app works correctly.	Technology Performance
37	Functions are correctly retrieved after events external to the app occur.	Technology Performance
38	The app uses mobile device resources efficiently (eg, battery, CPU, memory, data, network, etc), giving it a level of performance high enough not to interfere with the user’s experience.	Technology Performance
39	The app has a data recovery system in case of loss.	Technology Performance

## Results

### Overview

The results are organized according to the 3 research questions and the corresponding methodological components of the study. We first describe the mobile health apps identified through organizational recommendations and the scientific literature. We then summarize the empirical evidence available for these apps, including an assessment of the risk of bias and quality of these studies. Finally, we present the exploratory accessibility and functionality appraisal and the app quality assessment of the identified apps.

### Mobile Health Apps Identified Through Organizational Recommendations

After combining and deduplicating apps identified through organizational recommendations and the scientific literature, 19 unique mobile health apps were included in the review. Fifteen apps were identified through organizational recommendations and 11 were evaluated in the empirical studies included in the qualitative synthesis. These counts should not be summed because 7 apps were identified through both sources, 8 were identified only through organizational recommendations, and 4 were identified only through empirical studies. Apps were counted as unique when they represented distinct apps after comparison by app name, developer, intended function, and platform availability. Among the 15 apps identified through organizational recommendations, all were freely available, and most (14/15, 93%) were compatible with both iOS and Android platforms. Across the 19 unique apps included in the review, none specifically targeted psychological functioning or mental health concerns. Six apps (ie, Medisafe, MyFitnessPal, SACYL Cita Previa, Salud Informa, SCSalud, and SESCAM) were recommended by both Fundación ONCE and the American Foundation for the Blind, whereas the remaining apps were endorsed by a single organization.

Apps identified only through the scientific literature were retained as additional unique apps when they represented distinct mobile apps not already captured through organizational recommendations. Across the 15 apps identified through organizational recommendations, 2 broad functional categories were identified. First, several apps supported health self-management or health-related information access, including medication scheduling, medication information retrieval, glucose monitoring, nutrition tracking, or physical activity support. These included apps with medication scheduling and reminders (eg, Medisafe and Alarma de Medicamentos), medication information access or voice-based output (eg, ScripTalk and Medicamento Accesible Plus), glucose monitoring or diabetes self-management functions (eg, Glucose Buddy Diabetes Tracker, OneTouch Reveal, DiabetesPal, and FreeStyle LibreLink), and nutrition or weight management support (eg, MyFitnessPal). Second, several apps primarily supported health care access or administrative functions, such as appointment scheduling, prescription management, or integration with regional public health systems (eg, SACYL Cita Previa, Salud Informa, SCSalud, SESCAM, GVA *+* Salut and Astursalud Cita Previa). These apps are linked to specific Spanish regional public health systems and are generally restricted to individuals receiving care within those systems. They are therefore not broadly available or directly transferable to users in other national or regional health care contexts. These administrative apps were explicitly identified through the organizational recommendation component and may provide meaningful access to appointments, prescriptions, health records, and other services within their intended local settings. However, they should be interpreted as a separate category of locally contextualized health care access tools rather than as equivalent to internationally available clinical self-management apps, such as medication management, glucose monitoring, or health information tools. The main features and functional categories of these apps are summarized in Table 2.

**Table 2.** Main features of mobile health apps identified through organizational recommendations for individuals who are blind or have low vision.

Mobile app	Functional category^a^	Organization	Operating system	Developer	Languages	Key features	Price	Downloads, n
Alarma de Medicamentos	Medication management	Fundación ONCE	Android/iOS	Aplicativos legais LTDA-ME	English, Spanish, Portuguese, French, German, Italian, Russian, Korean, Turkish, and others	Customizable medication reminders and dosage tracking	Free with in-app purchases	>1 million
Astursalud Cita Previa	Health care access or administrative	Fundación ONCE	Android	Gobierno del Principado de Asturias	Spanish	Manage primary care appointments within the Asturias (Spain) regional public health system	Free	76,000
DiabetesPal	Diabetes self-management	American Foundation for the Blind	Android/iOS	Pascal Freiburghaus	English and German	Diabetes management: track glucose, insulin, and meals; sync across devices via iCloud, Dropbox, or WebDAV	Free to download with in-app purchases	>50,000 (Android)
FreeStyle LibreLink	Glucose monitoring	Fundación ONCE	Android/iOS	Abbott Diabetes Care Inc	Multilingual (includes English, Spanish, French, Italian, and more)	Glucose monitoring with sensor technology; provides historical trends; compatible with LibreView platform	Free to download with in-app purchases	>1 million
Glucose Buddy Diabetes Tracker	Diabetes self-management	American Foundation for the Blind	Android/iOS	Azumio Inc	English	Logs blood glucose, meals, and medications; integrates with Apple Health and Dexcom devices; provides detailed reports and optional coaching	Free with in-app purchases	>1 million
GVA + Salut	Health care access or administrative	Fundación ONCE	Android/iOS	Generalitat Valenciana	Spanish and Catalan from València	Appointment scheduling, medical records, prescriptions, telemedicine, and access to services within the Valencian (Spain) regional public health system	Free	2.4 million
Medicamento Accesible Plus	Medication information access	Fundación ONCE	Android/iOS	ILUNION Accesibilidad, Fundación ONCE, Fundación Vodafone España, and Consejo General de Colegios Farmacéuticos.	Spanish	Accessible medication information (indications, dosage, and warnings); includes food intolerance, pregnancy safety, and doping risk	Free	>50,000 (Android)
Medisafe	Medication management	American Foundation for the Blind/ONCE	Android/iOS	Medisafe Inc	Available in multiple languages, including English, Spanish, French, German, and more	Pill reminders and medication scheduling, drug interaction alerts, appointment tracking, and MedFriend caregiver support	Free with in-app purchases	>10 million (Android)
MyFitnessPal	Nutrition or weight management	American Foundation for the Blind/ONCE	Android/iOS	MyFitnessPal Inc	English and additional languages for localized versions	Food and nutrition tracking, barcode scanner, and macro- and micronutrient analysis	Free with optional in-app purchases for premium features	>200 million (worldwide)
OneTouch Reveal	Glucose monitoring	American Foundation for the Blind	Android/iOS	LifeScan Inc	English, French, German, Italian, Spanish, Portuguese, Dutch, Czech, Japanese, Slovenian, Polish, and Vietnamese	Tracks blood sugar readings via integration with OneTouch Verio Reflect and Verio Flex meters, allows manual data entry, and provides visual summaries, cloud synchronization, and web integration	Free	>1 million
SACYL Cita Previa	Health care access or administrative	American Foundation for the Blind/ONCE	Android/iOS	Junta de Castilla y León	Spanish	Manage, book, consult, and cancel appointments at health centers within the Castilla y León region (Spain); locate health centers; and manage multiple health cards	Free	>1 million
Salud Informa	Health care access or administrative	American Foundation for the Blind/ONCE	Android/iOS	Gobierno de Aragón	Spanish	Manage medical appointments, view treatment sheets, receive notifications, and manage health cards within the Aragón (Spain) regional public health system	Free	No data
SCSalud	Health care access or administrative	American Foundation for the Blind/ONCE	Android/iOS	Gobierno de Canarias	Spanish	Appointment management, medical record consultation, and health card information within the Canary Islands (Spain) regional public health system	Free	>500,000
SESCAM	Health care access or administrative	American Foundation for the Blind/ONCE	Android/iOS	Castilla-La Mancha Health Service	Spanish	Manage medical appointments, access health reports, and use personalized services within the Castilla-La Mancha (Spain) regional public health system	Free	>100,000
ScripTalk	Medication information access	World Blind Union	Android/iOS	En-Vision America Inc	Afrikaans, German, Amharic, Armenian, Bengali, Burmese, Bulgarian, Cambodian, Catalan, Czech, Chinese, Korean, Croatian, Danish, Slovak, Slovenian, Estonian, Filipino, Finnish, French, Greek, Haitian, Hebrew, Hindi, Hungarian, Indonesian, English, Italian, Japanese, Laotian, Latvian, Lithuanian, Malay, Marathi, Dutch, Nepali, Norwegian Bokmal, Pashto, Persian, Polish, Portuguese, Romanian, Russian, Serbian, Somali, Spanish, Swahili, Swedish, Thai, Turkish, Ukrainian, Vietnamese, and Arabic	Audio descriptions of prescription labels via RFID^b^ technology; reads drug name, dosage, and warnings	Free	>1000 (Android; no data for iOS)

a Functional categories distinguish apps primarily intended for health self-management or health-related information access from apps primarily intended to support health care access or administrative functions.

b RFID: radio frequency identification.

### Studies on Health-Related Apps in Scientific Databases

The database search was conducted in 2 phases. The initial search covered records published through December 2025 in ScienceDirect, PubMed or MEDLINE, and other NCBI resources. In April 2026, the search was updated across these 3 databases and expanded to include Scopus, Web of Science, and the ACM Digital Library; all 6 databases were searched from inception through April 2026. Across these database searches, 163 records were identified. In addition, 3 papers were identified through manual reference screening, yielding a total of 166 records. After removing 17 duplicates, 149 titles and abstracts of papers were screened. Of these, 101 were excluded, leaving 48 papers for full-text review. Following eligibility assessment, 14 studies were included in the qualitative synthesis.

An additional expanded search was also conducted across 6 databases to further examine the coverage of the search strategy: ScienceDirect, PubMed or MEDLINE, PubMed Central, Scopus, Web of Science, and the ACM Digital Library. This expanded search retrieved 1371 records: ScienceDirect (n=222), PubMed or MEDLINE (n=22), PubMed Central (n=54), Scopus (n=579), Web of Science (n=158), and ACM Digital Library (n=336). After duplicate removal and initial screening, 40 papers were selected for further review. Four papers underwent full-text eligibility assessment; however, none met the inclusion criteria. Therefore, the expanded search did not change the final number of included studies. Figure 1 presents the PRISMA flow diagram illustrating the paper selection process.

**Figure 1.**
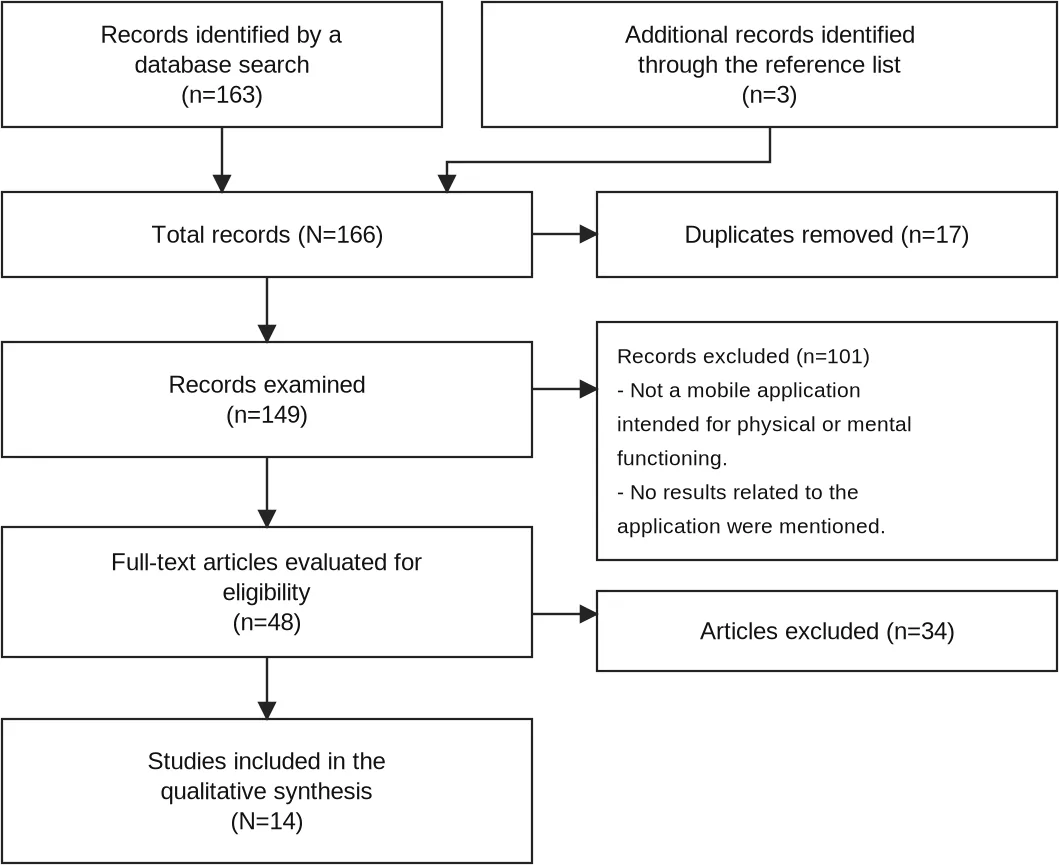
PRISMA (Preferred Reporting Items for Systematic Reviews and Meta-Analyses) flowchart illustrating the main study selection process for health-related mobile apps targeting individuals who are blind or have low vision.

The 14 empirical studies examined 11 different mobile health apps identified as relevant to individuals who are blind or have low vision, most often in the context of medication management, diabetes, hypertension, nutrition, or physical activity. However, the population focus of the evidence varied. Some studies specifically involved participants who were blind or had low vision, whereas others evaluated apps recommended for, or potentially relevant to, blind and low-vision users but were conducted in broader clinical or general populations where visual impairment status was not reported. Not all apps recommended by organizations had corresponding scientific evidence, and conversely, not all apps identified in the scientific literature were endorsed by organizations.

These studies used a range of methodologies: cross-sectional designs (6/14, 42%), observational studies (6/14, 42%), and randomized clinical trials (2/14, 14%). Most studies were conducted in the United States (6/14, 42%). Table 3 provides an overview of the 14 studies and the 11 apps evaluated. Because the included studies differed substantially in population focus and outcome type, Table 3 distinguishes between studies involving blind or low-vision participants and studies conducted in broader clinical or general populations where visual impairment status was not reported, and between studies evaluating objective or clinical outcomes and those focused primarily on usability, feasibility, satisfaction, or accessibility-related outcomes. Most of the identified apps were developed specifically to support medication management and access to pharmaceutical information. Despite geographic diversity, several core features were common: (1) voice output for medication identification and instructions; (2) barcode or Global Trade Item Number scanning to recognize medication packaging; (3) accessible search interfaces, usable by text, voice, or image; and (4) reminder systems for dosing and medication refills. These apps were primarily developed for Android platforms and followed user-centered design principles. Many included adjustable user interfaces (eg, text size, spoken descriptions, and voice navigation). Overall, the evidence was heterogeneous in both population focus and outcome type. Studies involving blind or low-vision participants primarily reported findings related to usability, feasibility, medication information access, perceived autonomy, or satisfaction with apps such as ScripTalk, MyPills, FarmaceuticApp, and Ru Tan Ya [5,44,45]. Studies conducted in broader clinical or general populations reported more objective or large-scale outcomes, including medication adherence and blood pressure control for Medisafe [5,46], glycemic metrics for FreeStyle LibreLink [47] and OneTouch Reveal [48,49], weight management behavior for MyFitnessPal [50], and dietary assessment accuracy for MyFitnessPal [51]. However, findings from broader populations should be interpreted as indirectly relevant to blind and low-vision users unless visual impairment status was specifically assessed. In addition, some barriers were noted. For example, the study of Medicamento Accesible Plus highlighted low digital literacy among older adults as a barrier to effective app use [52].

**Table 3.** Overview of empirical studies identified on mobile health apps identified as relevant to individuals who are blind or have low vision by population relevance and outcome type in chronological order of publication.

Authors (year), country	Purposes of the study	Apps	Principal functions	Population relevance to blind or low-vision users^a^	Languages	Sample of study	Type of study	Technical characteristics	Outcome type	Results
Spektor et al (2015) [53], United States	Evaluate effectiveness of ScripTalk for reading prescription labels	ScripTalk	Reads prescription labels aloud	Direct evidence: BLV^b^-specific sample	English	12 veterans with visual impairments	Field study	Allows iOS devices with Near Field Communication capability to read ScripTalk Talking Labels.	Medication access and adherence-related outcome	Improved treatment adherence and independence in medication use
Bee et al (2016) [54], Singapore	To evaluate the app for insulin use in insulin-naive patients with type 2 diabetes	DiabetesPal	Insulin adjustment based on glycemic goals	Indirect evidence: broader clinical population; BLV status not reported	English	62 participants (32 intervention, 30 control); type 2 adult patients with diabetes	Pilot randomized clinical trial	Diabetes data recording (eg, glucose, insulin, medication, blood pressure, etc); graph generation	Insulin titration feasibility and safety	Safe and feasible for insulin titration; no severe hypoglycemia recorded
Morawski et al (2018) [46], United States	To evaluate Medisafe’s impact on antihypertensive adherence and blood pressure control	Medisafe	Medication reminders, adherence reports, and caregiver support	Indirect evidence: broader clinical population; BLV status not reported	17 languages, including Spanish, English, Arabic, Danish, Dutch, Finnish, French, German, Hebrew, Italian, Japanese, Korean, Portuguese, Russian, Simplified Chinese, and Turkish	412 adults with uncontrolled hypertension	Randomized clinical trial	Adherence tracking; MedFriend support; multicondition tracking	Medication adherence and blood pressure control	Improved adherence significantly compared with the control group
González Oñate et al (2018) [52], Spain	Evaluate accessibility of medication information for older adults	Medicamento Accesible Plus	Search and read medicine information	Indirect evidence: general older adult population; BLV status not reported	Spanish	Older adults in Spain	Descriptive study	Barcode scanning; health database access	Accessibility and digital literacy	Identified digital divide, highlighting the need for promoting digital literacy
Gordon et al (2019) [55], United States	To analyze weight loss goal setting on MyFitnessPal	MyFitnessPal	Diet and exercise tracking tool	Indirect evidence: general app-user population; BLV status not reported	23 languages, including Spanish, English, French, German, Italian, Portuguese, Swedish, Danish, Norwegian, Bokmal, Dutch, Russian, Korean, Japanese Simplified, Chinese Traditional, Chinese Filipino, Indonesian, Malay, Polish, and Turkish	1.4 million users	Observational study	Meal planning; activity tracking; goal customization	Weight management behavior	Effective tool for behavioral change; consistent users had better weight loss outcomes
Nedovic et al (2019) [44], Switzerland	To examine how the app can support medication management for blind and visually impaired people	MyPills	Identification of medication packages, voice output of drug name and intake instructions, and voice output of package inserts	Mixed evidence: sample included blind participants and sighted participants	Different languages (not specified)	Two blind and 4 sighted individuals (aged 30‐70 years)	Cross-sectional study	Reminders for drug intake and reordering; audio access to package inserts; RFID^c^ scanning with GTIN^d^ support	Usability and medication management support	Users quickly understood the app; blind users found package scanning useful
Shade et al (2019) [50], United States	To explore usability and usefulness of Medisafe among older adults living in rural communities	Medisafe	Medication reminders	Indirect evidence: general older adult population; BLV status not reported	17 languages, including Spanish, English, Arabic, Danish, Dutch, Finnish, French, German, Hebrew, Italian, Japanese, Korean, Portuguese, Russian, Simplified Chinese, and Turkish	13 older adults residing in rural communities	Feasibility study	Adherence tracking; MedFriend support; multicondition tracking	Usability, usefulness, and feasibility	No usability issues, useful for medication tracking and adherence
Madrigal-Cadavid et al (2020) [5], Colombia	To design and develop a mobile drug information app for individuals with visual disabilities	FarmaceuticApp	Medication search (barcode, voice, or text command), access medication information, and nearby pharmacies/hospitals locator	Direct evidence: BLV-specific sample	Spanish	48 participants with visual disabilities (age range: 18‐60 years)	Cross-sectional descriptive study	Voice descriptions; scalable fonts; configurable accessibility settings; counterfeit detection; prescription history	User satisfaction and perceived usefulness	Rated 4 to 5 on a 1 to 5 numerical rating scale; all participants stated they would use it for medication adherence
Nimmolrat et al (2021) [45], Thailand	To analyze, design, and develop a pharmaceutical app for users with visual impairment and test usability	Ru Tan Ya	Search medicinal information, timer, map to pharmacies, medication history	Direct evidence: BLV-specific sample	Thai	60 volunteers	Experimental study	Touch screen; voice user interface design; slide rule and navigation techniques functions; optimized text or image access	Usability and satisfaction	Usability was low but most users were satisfied
Kao et al (2022) [56], United States	To evaluate glucose monitoring effectiveness using the app	FreeStyle LibreLink	Blood glucose monitoring and trend analysis	Indirect evidence: broader diabetes population; BLV status not reported	English	14,000 FreeStyle Libre users; patients with type 1 and 2 diabetes	Observational study	History view; blood glucose alarms; diet, insulin use, and exercise tracking; share data with doctor	Glucose metrics	Statistically significant improvements in blood glucose metrics compared with traditional reader users
Grady et al (2023) [47], United States– United Kingdom	To evaluate Bluetooth meter (OneTouch Verio Reflect) together with a mobile app (OneTouch Reveal) for diabetes control	OneTouch Reveal	Glucose monitoring, trend alerts, health data logging	Indirect evidence: broader diabetes population; BLV status not reported	12 languages, including Spanish, German, Czech, Slovenian, French, English, Italian, Japanese, Dutch, Polish, Portuguese, and Vietnamese	55,000 users; patients with type 1 and 2 diabetes	Retrospective observational study	Pattern detection; step tracking; glucose reports	Glycemic outcomes	Statistically significant improvements in glycemia: fewer highs or lows; better average blood glucose levels
Merino et al (2023) [48], different countries	To evaluate OneTouch Reveal (OTR Pro) for remote diabetes management during the COVID-19 pandemic	OneTouch Reveal	Monitoring glucose levels, reminders, track health data	Indirect evidence: broader diabetes population; BLV status not reported	12 languages, including Spanish, German, Czech, Slovenian, French, English, Italian, Japanese, Dutch, Polish, Portuguese, and Vietnamese	1500 users during virtual consultations	Descriptive observational study	Detects blood glucose patterns; tracks daily steps; creates reports for therapeutic decisions	Clinical practice or diabetes management support	Facilitated clinical practice; ease of secure data sharing; enhanced therapeutic decisions
Banal et al (2024) [49], Philippines	To evaluate validity and intercoder reliability of MyFitnessPal for dietary assessment	MyFitnessPal	Diet and exercise tracking tool	Indirect evidence: broader diabetes population; BLV status not reported	21 languages, including Spanish, English, French, German, Italian, Portuguese, Swedish, Danish, Norwegian, Bokmal, Dutch, Russian, Korean, Japanese Simplified, Chinese Traditional, Chinese Filipino, Indonesian, Malay, Polish, and Turkish	37 Filipino adults with obesity	Observational study	Meal planning; activity tracking; dietary intake estimations	Dietary assessment validity and reliability	Useful for diet tracking; some limitations in nutritional estimate accuracy
Choi et al (2025) [51], United States	Develop an accessible conversational mHealth app (Personalized and Conversational Health Agent) to support physical activity in blind and low-vision individuals	Personalized and Conversational Health Agent	Personalized and Conversational Health Agent is a conversational, voice-based health assistant that provides personalized recommendations for physical activity, enables self-monitoring of health metrics (steps and heart rate), and facilitates interactive communication via a voice user interface	Direct evidence: BLV-specific expert evaluation	English with voice-based natural language interaction (ie, via a conversational agent using a large language model)	Two blind accessibility experts evaluated the prototype	Design and development study with qualitative expert evaluation	Uses iOS with large language model integration; voice user interface; multimodal feedback (audio and haptic via Apple Watch); uses physical activity assessment and activity guidelines for Americans to provide physical activity recommendations via an algorithm	Prototype accessibility and expert evaluation	Successful development of a functional prototype with accessible user interface, effective voice interaction, and multimodal feedback; includes 2 experts to test the prototype; limitations identified included response latency, a lack of personalized physical activity feedback, and the need for clearer goal-setting explanations

a “Direct evidence” refers to studies that specifically included blind or low-vision participants or expert evaluators. “Mixed evidence” refers to studies including both blind or low-vision and sighted participants. “Indirect evidence” refers to studies evaluating apps identified as relevant to blind or low-vision users but conducted in broader clinical or general populations where visual impairment status was not reported.

b BLV: blind or low vision.

c RFID: radio frequency identification.

d GTIN: Global Trade Item Number.

### Assessment of Bias and Quality of Evidence

The interrater reliability in the assessment of methodological quality was high, with 98% agreement between reviewers across all included studies. This high level of agreement reflects the use of a structured appraisal framework with clearly defined criteria, as well as prior calibration between reviewers before the formal assessment. Minor discrepancies, mainly due to different interpretations of the criteria, were resolved through discussion, without the need to involve a third reviewer. A considerable proportion of the studies included in the review presented a moderate or high risk of bias, and out of 14 studies, only 6 (42%) met the criteria for good methodological quality: DiabetesPal [49,54,55], Medisafe [52], MyFitnessPal [50,51], FreeStyle LibreLink [47], and OneTouch Reveal [48]. Most papers reported a clear objective, defined population, and inclusion and exclusion criteria [47,48,56]. However, many did not report participation rates or whether assessors were blinded [45,52]. Table 4 provides a summary of this information. Table 4 should be interpreted as a structured overview of methodological quality across NIH criteria. The overall quality rating reflects the number of criteria met, whereas “Cannot determine” indicates insufficient information to judge an item and “Not applicable” indicates that the criterion was not relevant to the study design.

**Table 4.** Methodological quality of studies—the National Institutes of Health Quality Assessment Tool^a^.

Authors (year)	1	2	3	4	5	6	7	8	9	10	11	12	13	14	Quality rating^b^
Banal et al (2024) [49]	Yes	Yes	Yes	Yes	Yes	Yes	Yes	Yes	Yes	Yes	Yes	Yes	Yes	Yes	Good
Bee et al (2016) [54]	Yes	Yes	CD^c^	Yes	Yes	Yes	Yes	Yes	Yes	Yes	Yes	CD	Yes	CD	Good
Choi et al (2025) [51]	Yes	Yes	NA^d^	Yes	Yes	NA	NA	NA	CD	NA	CD	NA	NA	NA	Poor
González Oñate and Fanjul Peyró (2018) [52]	Yes	Yes	No	Yes	Yes	Yes	Yes	Yes	Yes	Yes	Yes	No	NA	No	Fair
Gordon et al (2019) [55]	Yes	Yes	Yes	Yes	Yes	Yes	Yes	Yes	Yes	Yes	Yes	Yes	Yes	Yes	Good
Grady et al (2023) [47]	Yes	Yes	CD	Yes	Yes	Yes	Yes	Yes	Yes	Yes	Yes	CD	Yes	CD	Good
Kao et al (2022) [56]	Yes	Yes	Yes	Yes	Yes	Yes	Yes	Yes	Yes	Yes	Yes	NA	Yes	Yes	Good
Madrigal-Cadavid et al (2020) [5]	Yes	Yes	Yes	Yes	Yes	NA	NA	NA	Yes	NA	Yes	CD	NA	NA	Fair
Merino et al (2023) [48]	Yes	Yes	CD	Yes	Yes	Yes	Yes	Yes	Yes	Yes	Yes	CD	CD	CD	Fair
Morawski et al (2018) [46]	Yes	Yes	Yes	Yes	Yes	Yes	Yes	Yes	Yes	Yes	Yes	No	Yes	Yes	Good
Nedovic et al (2019) [44]	Yes	Yes	CD	Yes	No	NA	NA	NA	Yes	NA	Yes	No	Yes	No	Poor
Nimmolrat et al (2021) [45]	Yes	Yes	CD	Yes	Yes	NA	NA	NA	Yes	NA	Yes	CD	NA	NA	Poor
Shade et al (2019) [50]	Yes	Yes	No	Yes	Yes	Yes	Yes	Yes	Yes	No	Yes	No	Yes	No	Fair
Spektor et al (2015) [53]	Yes	Yes	CD	Yes	Yes	Yes	Yes	Yes	Yes	Yes	Yes	No	NA	CD	Fair

a These items are as follows: 1=study objectives stated, 2=study population defined, 3=eligible participation rate (at least 50%), 4=participant selection and inclusion and exclusion criteria uniformity, 5=sample size sufficient and/or described, 6=exposure measured prior to outcome, 7=sufficient time frame for association between exposure and outcome, 8=inclusion exposure level, 9=exposure measures valid and reliable, 10=multiple exposure measurements, 11=outcome measures valid and reliable, 12=outcome assessors blinded, 13=Loss to follow-up postbaseline (20% or less), and 14=confounders measured and adjusted between exposure and outcome.

b Quality ratings were derived using the National Institutes of Health Quality Assessment Tool. Overall ratings were based on the total number of criteria met: poor=0‐6 “Yes” responses, fair=7‐10 “Yes” responses, and good=11‐14 “Yes” responses.

c CD: cannot determine (insufficient information available to judge the criterion).

d NA: not applicable (criterion not relevant to the study design).

### Accessibility, Functionality, and Quality of the Identified Mobile Apps

We conducted an exploratory user-informed accessibility appraisal of the identified apps using the WCAG 2.1 principles: Perceivable, Operable, Understandable, and Robust. These ratings should be interpreted as illustrative user-informed observations rather than as formal accessibility certification, psychometric assessment, or standardized measurement outcomes. The appraisal suggested variability in accessibility across the apps. Within this exploratory user-informed appraisal, MyPills received the most favorable accessibility profile, obtaining “High” ratings across all 4 WCAG 2.1 [43] principles. Participants highlighted its compatibility with screen readers, clear instructions, and reliable performance across devices. In contrast, 6 apps (ie, Astursalud Cita Previa, Glucose Buddy Diabetes Tracker, SESCAM, SCSalud, SACYL Cita Previa, and MyFitnessPal) received “Low” ratings on most or all WCAG principles within this exploratory appraisal. Common issues included poor support for assistive technologies, visually cluttered interfaces without alternatives, and navigation challenges for screen reader users. Examples of accessibility barriers identified during the appraisal included unlabeled controls, insufficient clarity of instructions, inconsistent navigation flows, and limited compatibility with screen-reader interaction. Conversely, apps with clearer menu structures, accessible labeling, and more predictable navigation were judged more favorably. Of the mobile apps identified, 2 (ie, FarmaceuticApp and Ru Tan Ya) could not be downloaded or assessed because they were not available for download in the app stores at the time of assessment. Accessibility ratings of the remaining apps are summarized in Table 5.

**Table 5.** Exploratory user-informed accessibility appraisal of mobile apps for users who are blind or have low vision based on the WCAG^a^ 2.1 principles^b^.

Name of app	Principle 1: Perceivable	Principle 2: Operable	Principle 3: Understandable	Principle 4: Robust
Alarma de Medicamentos	High	High	High	Moderate
Astursalud Cita Previa	Low	Low	Low	Low
DiabetesPal	Moderate	Moderate	Moderate	Low
FreeStyle LibreLink	Moderate	Moderate	Moderate	Low
Glucose Buddy Diabetes Tracker	Low	Moderate	Low	Low
GVA + Salut	Moderate	Moderate	Moderate	Low
Medicamento Accesible Plus	Moderate	Moderate	Moderate	Low
Medisafe	High	High	High	Moderate
MyFitnessPal	Low	Moderate	Low	Low
MyPills	High	High	High	High
OneTouch Reveal	Moderate	High	Moderate	Moderate
Personalized conversational health agent	High	Moderate	Moderate	Low
SACYL Cita Previa	Low	Low	Low	Low
Salud Informa	Moderate	Low	Moderate	Low
SCSalud	Low	Low	Low	Low
SESCAM	Low	Low	Low	Low
ScripTalk	High	High	High	Moderate

a WCAG: Web Content Accessibility Guidelines.

b Ratings were derived from an exploratory user-informed appraisal involving 4 participants (2 blind and 2 with low vision) and should not be interpreted as formal accessibility certification, psychometric assessment, or standardized measurement outcomes.

App quality was assessed using the MAG [34], which includes 39 criteria covering usability, privacy, security, safety, appropriateness, and technology performance. The appraisal showed substantial variability across apps. Medisafe scored highest, meeting 34 of 39 criteria, whereas SACYL Cita Previa scored lowest, meeting 8 of the 39 criteria. Table 6 presents a summary of the MAG total score and the descriptive score band assigned to each app for this review. Higher total scores indicate that a greater number of quality criteria were met, whereas lower scores reflect more limited compliance across MAG domains. The complete item-level MAG appraisal is provided in Multimedia Appendix 4.

**Table 6.** Summary of app-quality appraisal using the Mobile App Development and Assessment Guide (MAG).

App	MAG total score of 39, n^a^	Descriptive MAG-based interpretation^b^
Medisafe	34	Higher app quality
Medicamento Accesible Plus	33	Higher app quality
ScripTalk	32	Higher app quality
OneTouch Reveal	31	Higher app quality
FreeStyle LibreLink	29	Higher app quality
Glucose Buddy Diabetes Tracker	29	Higher app quality
MyFitnessPal	28	Higher app quality
MyPills	27	Higher app quality
Salud Informa	25	Moderate app quality
DiabetesPal	24	Moderate app quality
GVA + Salut	22	Moderate app quality
SCSalud	18	Lower app quality
SESCAM	18	Lower app quality
Astursalud Cita Previa	16	Lower app quality
Alarma de Medicamentos	13	Lower app quality
Personalized Conversational Health Agent	12	Lower app quality
SACYL Cita Previa	8/39	Lower app quality

a The MAG includes 39 criteria. Higher scores indicate that a greater number of app-quality criteria were met.

b Descriptive score bands were created for this review because the MAG does not provide predefined cutoff points for interpreting total scores: higher app quality ≥27/39 criteria met, moderate app quality 20-26/39 criteria met, and lower app quality ≤19/39 criteria met. These categories were used only to aid interpretation of the descriptive MAG scores and should not be interpreted as validated MAG thresholds. The full item-level MAG appraisal is provided in Multimedia Appendix 4.

## Discussion

### Principal Findings

This systematic review examined the current landscape of mobile health apps intended to support the physical and psychological functioning of individuals who are blind or have low vision. Overall, the findings suggest that this field remains limited in scope, uneven in accessibility and quality, and only partially supported by empirical evidence.

Three main findings emerged. First, the number of identified apps was small, and only a subset had been evaluated in peer-reviewed studies. Second, the available apps focused primarily on physical functioning, particularly medication management and health data tracking, whereas no apps specifically targeting psychological functioning were identified. Third, accessibility and app quality varied considerably, with only a small number of apps performing well across both WCAG and MAG criteria. These findings are broadly consistent with a recent systematic review on the usability of mobile apps for individuals with visual impairments [29]. Although that review did not focus specifically on health-related apps or on physical and psychological functioning, it similarly concluded that the usability of mobile apps for visually impaired users remains limited due to device-related barriers (eg, camera-based interfaces) and user interaction challenges (eg, difficulties interpreting graphical user interfaces) [29].

With respect to the first research question, the apps identified through organizational recommendations, and the systematic search of scientific literature reflected a relatively narrow functional landscape. Most supported medication use, medication information access, glucose monitoring, nutrition tracking, or physical activity support. A separate subset of apps primarily supported health care access or administrative functions, such as appointment scheduling, prescription management, or access to regional health system information. These latter tools may be useful for navigating health care services, particularly in local contexts, but they are functionally distinct from clinical self-management apps such as medication management, glucose monitoring, nutrition tracking, or physical activity tools. Overall, the identified apps represent a restricted subset of the possible ways in which mobile health apps might support functioning in individuals who are blind or have low vision. As a result, they do not reflect a broader conceptualization of functioning that includes psychological well-being, self-efficacy, autonomy, and participation in daily life.

Importantly, the characterization of this landscape as limited should not be understood as a simple consequence of the number of apps identified or of the inclusion of geographically restricted administrative tools. Rather, it reflects the narrow range of functions addressed across the identified apps, the limited number supported by population-specific empirical evidence, the variability observed in accessibility and app quality, and the absence of apps primarily addressing psychological functioning.

A particularly important finding was the absence of apps specifically targeting psychological functioning. This should be interpreted cautiously, as it reflects an absence of identified apps rather than definitive evidence that none exists. Some apps may include psychological or mental health–related components; however, within the scope of this review, we did not identify apps primarily designed for, or empirically evaluated in, blind and low-vision populations with a specific focus on psychological or mental health functioning. Nonetheless, this gap is clinically meaningful given the elevated burden of depression, anxiety, and psychological distress in individuals who are blind or have low vision [19]. This observation is consistent with Inal and colleagues [57], who reviewed 42 usability studies of mobile mental health technologies and found that none included users with visual impairments. Taken together, these findings suggest that psychological functioning remains insufficiently addressed in both app development and research for this population. By contrast, app-based interventions addressing pain-related symptoms and quality of life have been developed for other chronic pain populations. For example, Fibroline incorporates a self-administered cognitive behavioral intervention designed for young people with fibromyalgia or chronic widespread pain, illustrating how psychological and symptom management components can be integrated into a mobile health app outside the context of blindness and low vision [24,58].

With respect to the second research question, the empirical evidence base was limited in both size and scope. In this review, “evidence” refers to the existence of empirical studies examining an app and should not be interpreted as formal validation in a psychometric, regulatory, or certification sense. Only a minority of identified apps had been examined in empirical studies, and the methodological quality of those studies was variable. Importantly, the available evidence differed in its relevance to blind and low-vision users and in the type of outcomes assessed. Some studies directly involved participants who were blind or had low vision, whereas others evaluated apps recommended for this population but did so in broader clinical or general populations where visual impairment status was not reported. Therefore, evidence of effectiveness or usability in general populations should be interpreted as indirectly relevant rather than as population-specific evidence for blind and low-vision users. Importantly, the available evidence also differed according to the type of outcomes that were assessed. Some studies reported objective or clinical outcomes, including medication adherence and blood pressure control, glucose metrics, weight management behavior, or dietary assessment accuracy. Other studies focused primarily on usability, feasibility, acceptability, satisfaction, medication information access, or perceived usefulness. Consequently, the current evidence base is better understood as comprising 2 complementary forms of evidence: studies evaluating objective or clinical outcomes, predominantly conducted in broader populations, and studies examining usability, accessibility, or user experience, more commonly conducted with participants who were blind or had low vision. This distinction is important because the existence of an empirical study does not necessarily imply demonstrated clinical effectiveness, and findings from broader populations should not be interpreted as direct evidence of effectiveness, accessibility, or usability for blind and low-vision users. Studies conducted in broader clinical or general populations examined objective or clinical outcomes for Medisafe [46], including medication adherence and blood pressure control; FreeStyle LibreLink [56] and OneTouch Reveal [47,48], including glycemic metrics; and MyFitnessPal [49,55], including weight management behavior and dietary assessment accuracy. By contrast, studies involving blind or low-vision participants more commonly examined usability, feasibility, medication information access, satisfaction, perceived autonomy, or prototype accessibility for apps such as ScripTalk [53], MyPills [44], FarmaceuticApp [5], Ru Tan Ya [45], and PCHA [51]. Accordingly, the literature provides some evidence of clinical or behavioral benefit for selected apps in broader populations and some direct evidence of usability or perceived value among blind or low-vision users but very limited evidence combining population-specific evaluation with objective health-related outcomes. The review also identified only partial overlap between apps recommended by organizations and apps supported by empirical evidence. That is to say, several apps endorsed by organizations lacked corresponding scientific evaluation, whereas some apps described in the literature were not recommended by organizations. This discrepancy is important because organizational recommendations may shape what users, families, and clinicians perceive as credible or useful. The findings therefore highlight the need for closer alignment between research, practice, and dissemination processes so that recommended tools are not only accessible but also supported by evidence.

With respect to the third research question, accessibility and app quality were inconsistent across the identified apps. Only a small number of apps appeared to perform well across the exploratory WCAG-informed appraisal and the MAG assessment, whereas others showed substantial limitations. In the exploratory user-informed appraisal, some apps endorsed by organizations appeared to present potential accessibility barriers, including unlabeled graphical elements, limited screen reader compatibility, and complex navigation structures. These observations should be interpreted cautiously because they were derived from a small exploratory appraisal rather than from a formal accessibility validation study. Nevertheless, they are consistent with previous work in accessibility, human-computer interaction, and assistive technology, which has highlighted persistent problems related to interface complexity, visually dependent interaction models, and inadequate compatibility with assistive tools [29,59-61]. Our findings extend that literature by showing that these barriers remain highly relevant even in apps with apparent health value or organizational endorsement.

The user-informed accessibility appraisal also suggests that technical accessibility alone may not be sufficient to ensure meaningful use. Even when apps were theoretically compatible with assistive features, their practical value could still be limited by poor onboarding, inadequate support, unfamiliar interaction patterns, or broader digital literacy barriers. Accessibility, therefore, should not be understood solely as technical compliance but also as the extent to which an app can be used effectively in real-world contexts by its intended users.

At the same time, some apps demonstrated comparatively strong accessibility and overall quality. For example, within the exploratory WCAG-informed appraisal, MyPills received the most favorable accessibility profile, obtaining “High” ratings across all 4 WCAG principles, whereas Medisafe achieved the highest MAG score (34/39), suggesting particularly strong performance in broader app quality domains. More generally, the studies with the strongest designs reported improvements in medication outcomes, such as medication adherence, self-management, and user satisfaction. However, these findings should be interpreted cautiously, given the limited number of studies, their relatively small samples, and the generally high digital literacy of participants. By contrast, many other apps received low accessibility or quality ratings, and the methodological quality of the supporting studies was mixed overall. Several apps endorsed by organizations still showed substantial accessibility limitations across 1 or more WCAG domains and performed less well on the MAG appraisal. For example, despite being recommended by ONCE and the American Foundation for the Blind, MyFitnessPal relied heavily on visually structured content, complex menu hierarchies, and limited screen reader compatibility, which may reduce usability for individuals who are blind or have low vision. This illustrates a potentially important mismatch between organizational endorsement and real-world accessibility. More broadly, even apps that appeared technically accessible could still be difficult to use in practice because of limited onboarding, insufficient guidance, low awareness, and digital literacy barriers. Taken together, these findings suggest that technical accessibility alone is insufficient; effective dissemination, onboarding, and real-world usability are also essential to achieve meaningful impact.

These findings have several important implications. From a clinical perspective, they suggest that only a small number of apps currently offer reasonably credible and accessible tools for supporting physical functioning, particularly medication self-management. Clinicians may wish to be aware of these options but should also recognize that endorsement or availability does not necessarily imply accessibility, quality, or evidence of benefit. At the same time, the absence of identified apps targeting psychological functioning leaves an important gap in the digital support currently available for this population. From a development, policy, and research perspective, the findings point to the need for more rigorous and transparent standards for app development, evaluation, and recommendation. Apps intended for individuals who are blind or have low vision should be developed through participatory and inclusive co-design processes involving end users, clinicians, and accessibility specialists, and should be evaluated not only for intended usefulness but also for real-world accessibility and usability. This perspective is consistent with recent developments reflected in the WCAG 3.0 Working Draft [62]. Although this document is not a ratified standard, it illustrates an ongoing shift from a predominantly technical, criteria-based approach toward a more flexible framework centered on users’ diverse accessibility needs. In addition, future research should prioritize apps that address psychological functioning in ways that are both accessible and clinically meaningful and should include larger and more diverse samples, stronger study designs, and outcomes that extend beyond feasibility or satisfaction to capture meaningful indicators of functioning. Greater attention should also be given to accessible onboarding, guidance, and support features to facilitate sustained real-world use. Finally, future work should examine how emerging assistive and AI-enabled functionalities can be integrated into mobile health apps without increasing complexity or introducing new accessibility barriers.

### Limitations

This review has several limitations that should be acknowledged. First, the identified studies were heterogeneous in design, geography, sample size, participant characteristics, and outcome measures, limiting comparability and precluding meta-analysis. In addition, although the present review included studies with qualitative and mixed methods components, it was not designed as a formal qualitative evidence synthesis. Applying frameworks such as those proposed in the Cochrane-Campbell Handbook for Qualitative Evidence Synthesis would entail a substantially different review design but may represent a valuable direction for future research focused on in-depth analysis of user experience and interaction design in this field. Second, restricting the search to English and Spanish publications may have excluded relevant studies in other languages, and despite a comprehensive search strategy, some studies may have been missed. In addition, some apps identified through the literature could not be directly assessed because they were not available for download at the time of review, which may introduce a degree of availability bias. Moreover, identifying apps through organizational recommendations may have introduced selection bias, as not all organizations publish or regularly update such recommendations. This process also introduced geographic and contextual limitations. Several organizationally identified apps were region-specific health care access or administrative tools linked to regional public health systems, which may limit their relevance to international readers and make them functionally distinct from clinical self-management apps. Therefore, the number and nature of identified apps should be interpreted in light of the sources consulted and the organizational recommendation process. Finally, the accessibility appraisal should be interpreted as a complementary and exploratory component intended to contextualize the review findings. It was not designed as a stand-alone usability study, formal accessibility validation, or psychometric assessment. Therefore, its findings should be understood as illustrative user-informed observations of potential accessibility barriers rather than as generalizable estimates of usability or definitive measurements of accessibility across the broader population of users who are blind or have low vision. However, its use is consistent with prior accessibility literature and with W3C guidance supporting the application of WCAG principles to mobile and nonweb software.

Despite the study’s limitations, this review shows that the mobile health apps identified through organizational recommendations and the scientific literature remain limited in scope, heterogeneous in function, uneven in accessibility and quality, and supported by a limited and heterogeneous empirical base. While a small number of apps appear promising, major gaps remain in accessibility, quality, and especially support for psychological functioning. Moving the field forward will require more than simply increasing the number of available apps. Future efforts should focus on developing apps that are accessible from the outset, rigorously evaluated, and developed in collaboration with the people they are designed to support.

## Supplementary material

10.2196/85809 Multimedia Appendix 1 Description of updated search.

10.2196/85809 Multimedia Appendix 2 Data extraction template.

10.2196/85809 Multimedia Appendix 3 Operationalization and scoring of Web Content Accessibility Guidelines (WCAG) 2.1 principles in the appraisal of mobile health apps.

10.2196/85809 Multimedia Appendix 4 Quality assessment of health-related mobile apps for individuals who are blind or have low vision, based on the Mobile App Development and Assessment Guide criteria.

10.2196/85809 Checklist 1 PRISMA checklist.

## Abbreviations

MAG: Mobile App Development and Assessment Guide
NCBI: National Center for Biotechnology Information
NIH: National Institutes of Health
ONCE: Spanish National Organization for the Blind
PRISMA: Preferred Reporting Items for Systematic Reviews and Meta-Analyses
W3C: World Wide Web Consortium
WCAG: Web Content Accessibility Guidelines
