# Supplementary material for: Mobile Health Apps for Individuals Who Are Blind or Have Low Vision: Systematic Review of Evidence, Accessibility, and App Quality
Source: JMIR Mhealth Uhealth. 2026 Nov 12;14:e85809. doi: 10.2196/85809 (PMC13759188; doi:10.2196/85809)
Supplement: Supplementary file 1 [file mhealth-v14-e85809-s001.docx]

Multimedia Appendix 1

Database-Specific Search Strategies for the Updated Search

# 1. Overview of the search strategy

A systematic literature search was conducted to identify empirical studies evaluating mobile health applications intended to support the physical and psychological functioning of individuals who are blind or have low vision.

The review included an initial search covering studies published up to December 2025 and an updated and expanded search conducted in April 2026 to improve coverage of interdisciplinary literature relevant to digital health, assistive technology, accessibility, and human-computer interaction.

The updated search was performed across six electronic databases: ScienceDirect, PubMed/MEDLINE, PubMed Central (PMC), Scopus, Web of Science, and the ACM Digital Library.

# 2. Search strategy development and refinement

Search strategies were adapted to the indexing system of each database and applied to title and abstract fields or their equivalents, including keywords in ScienceDirect when available.

Searches combined terms across two conceptual domains:

- Technology-related terms: "mobile app", "smartphone", and related mobile health descriptors.
- Population-related terms: "visually impaired", "visual disability", "blind", "blindness", "low vision", and related descriptors.

Boolean operators (AND/OR) were used to combine terms within and across these conceptual domains.

Initial searches yielded a high proportion of irrelevant results in several databases. An iterative refinement process was therefore implemented to improve specificity while preserving conceptual coverage. This process involved removing overly broad or ambiguous terms, replacing truncated terms that generated irrelevant results, restricting technology-related terms to more specific descriptors such as "mobile app" and "smartphone", and incorporating methodological terms such as "evaluation" and "empirical study" where appropriate.

# 3. Database-specific search strategies and records retrieved

| **Database** | **Search fields / filters** | **Records retrieved** |
| --- | --- | --- |
| ScienceDirect | Title, abstract, and author keywords | 222 |
| PubMed/MEDLINE | Title and abstract | 22 |
| PubMed Central (PMC) | Title and abstract | 54 |
| Scopus | Title, abstract, and keywords (TITLE-ABS-KEY) | 579 |
| Web of Science | Topic search (TS) | 158 |
| ACM Digital Library | Keyword search; filtered to research articles | 336 |

**Total records retrieved across all six databases: 1,371.**

## 3.1 ScienceDirect

Search fields: Title, abstract, and author keywords.

Final search string:

( "mobile app" OR "smartphone" ) AND ( "visually impaired" OR "visual disability" OR "blind" OR "low vision" OR "ocular" ) AND ( "evaluation" OR "empirical study" )

Records retrieved: n = 222.

## 3.2 PubMed/MEDLINE

Search fields: Title and abstract.

Final search string:

( "mobile app" OR "smartphone" ) AND ( "visually impaired" OR "visual disability" OR "blind" OR "blindness" OR "low vision" )

Records retrieved: n = 22.

## 3.3 PubMed Central (PMC)

Search fields: Title and abstract.

Final search string:

( "mobile app" OR "smartphone" ) AND ( "visually impaired" OR "visual disability" OR "blind" OR "blindness" OR "low vision" )

Records retrieved: n = 54.

## 3.4 Scopus

Search fields: Title, abstract, and keywords (TITLE-ABS-KEY).

An initial broad search combining mobile technology terms (e.g., app*, mobile, smartphone) and visual impairment terms (e.g., visually impaired, visual disability, blind*, low vision, ocular*, eye*) yielded a high number of irrelevant records. To improve specificity, the final search was restricted to more specific terms.

Final search string:

TITLE-ABS-KEY ( "mobile app" OR "smartphone" ) AND TITLE-ABS-KEY ( "visually impaired" OR "visual disability" OR "low vision" )

Records retrieved after refinement: n = 579.

## 3.5 Web of Science

Search fields: Topic search (TS).

A comparable combination of mobile technology and visual impairment terms was used. To improve specificity, the search strategy incorporated an additional assistance-related concept.

Final search string:

TS = ( "mobile app" OR "smartphone" ) AND TS = ( "visually impaired" OR "visual disability" OR "low vision" ) AND TS = ( "assistance" OR "assistive" OR "accessibility" )

Records retrieved: n = 158.

## 3.6 ACM Digital Library

Search fields: keyword-based search across database records.

Final search structure:

( "mobile app" OR "smartphone" ) AND ( "visually impaired" OR "visual disability" OR "blind" OR "blindness" OR "low vision" )

The initial keyword-based search yielded 537 records. Results were filtered by publication type to include research articles only, resulting in 336 records.

Records retained after filtering: n = 336.

# 4. Screening and eligibility process for the updated search

Following duplicate removal and initial screening, titles and abstracts of retrieved records were reviewed for relevance. Based on this process, 40 articles were selected for further review, focusing on studies addressing physical and mental health outcomes among individuals who are blind or have low vision.

Of these, four articles were identified as potentially eligible and underwent full-text review. None met the inclusion criteria. Reasons for exclusion included: focus outside the target health scope, duplicate publication, absence of relevant health outcomes, or absence of participants who were blind or had low vision.

The updated search did not identify additional studies meeting the inclusion criteria and therefore did not change the final set of included studies.

# 5. Additional search procedures

The database search was complemented by manual reference list screening of included studies and by app-name searches. In the app-name search, applications identified through organizational sources were searched individually using combinations of app names and population-related terms. These procedures were used to ensure that empirical studies on apps already recommended in practice were not overlooked and to complement the database-based identification of relevant studies.

# 6. Data extraction procedure

Data extraction was conducted using a structured template developed for this review. Extracted information included:

- Study characteristics: authors, year, country, and study design.
- Participant characteristics, including population and visual impairment status where reported.
- Mobile application characteristics: app name, purpose, platform, language, and target health domain.
- Type of intervention, assessment, or support provided by the application.
- Accessibility, usability, and health-related outcomes assessed.
- Key findings reported by each study.

Data extraction followed predefined criteria to ensure consistency and comparability across studies.
