# Supplementary material for: Mobile Health Apps for Individuals Who Are Blind or Have Low Vision: Systematic Review of Evidence, Accessibility, and App Quality
Source: JMIR Mhealth Uhealth. 2026 Nov 12;14:e85809. doi: 10.2196/85809 (PMC13759188; doi:10.2196/85809)
Supplement: Supplementary file 2 [file mhealth-v14-e85809-s002.docx]

| Study ID | Authors (Year) | Country | Population | Type of App | Study Design | Outcomes Assessed | Key Findings | Notes |
| --- | --- | --- | --- | --- | --- | --- | --- | --- |

**Multimedia Appendix 2**

**Data Extraction template**
