# Supplementary material for: Mobile Health Apps for Individuals Who Are Blind or Have Low Vision: Systematic Review of Evidence, Accessibility, and App Quality
Source: JMIR Mhealth Uhealth. 2026 Nov 12;14:e85809. doi: 10.2196/85809 (PMC13759188; doi:10.2196/85809)
Supplement: Supplementary file 3 [file mhealth-v14-e85809-s003.docx]

**Multimedia Appendix 3**

**Operationalization and Scoring of WCAG 2.1 Principles in the Appraisal of Mobile Health Applications**

Note. Accessibility was evaluated following WCAG 2.1 principles (POUR: Perceivable, Operable, Understandable, Robust). Each domain was rated using a 3-point scale: 0 = Not met, 1 = Partially met, 2 = Fully met. Ratings were based on structured observation of app interaction using assistive technologies (e.g., screen readers).

| Principle | Evaluation Focus | Operationalization | Scoring Criteria | Example (Compliant) | Example (Non-Compliant) |
| --- | --- | --- | --- | --- | --- |
| Perceivable | Content is accessible via sensory channels | Assessment of labels, screen reader output, and feedback | 0 = Not accessible; 1 = Partially labeled/inconsistent; 2 = Fully labeled and accessible | All buttons labeled and correctly announced | Icons without labels; screen reader cannot identify elements |
| Operable | Navigation and controls usable via assistive tech | Evaluation of navigation structure and gesture compatibility | 0 = Not operable; 1 = Partially operable; 2 = Fully operable | Logical navigation accessible via VoiceOver/TalkBack | Gesture-only navigation inaccessible with screen reader |
| Understandable | Interface and content are clear and predictable | Assessment of instructions, consistency, and flow | 0 = Confusing; 1 = Some clarity issues; 2 = Clear and consistent | Clear instructions and consistent layout | Ambiguous labels and inconsistent navigation |
| Robust | Compatibility with assistive technologies | Evaluation across accessibility settings and tools | 0 = Fails with assistive tech; 1 = Partial compatibility; 2 = Fully compatible | Works reliably with screen readers | Crashes or fails when assistive tech is enabled |
