# Supplementary material for: Mobile Health Apps for Individuals Who Are Blind or Have Low Vision: Systematic Review of Evidence, Accessibility, and App Quality
Source: JMIR Mhealth Uhealth. 2026 Nov 12;14:e85809. doi: 10.2196/85809 (PMC13759188; doi:10.2196/85809)
Supplement: Supplementary file 4 [file mhealth-v14-e85809-s004.docx]

**Multimedia Appendix 4**

**Quality assessment of health-related mobile applications for individuals who are blind or have low vision, based on the Mobile App Development and Assessment Guide (MAG) criteria**

| **Name of App** | **1^a^** | **2** | **3** | **4** | **5** | **6** | **7** | **8** | **9** | **10** | **11** | **12** | **13** | **14** | **15** | **16** | **17** | **18** | **19** | **20** |
| --- | --- | --- | --- | --- | --- | --- | --- | --- | --- | --- | --- | --- | --- | --- | --- | --- | --- | --- | --- | --- |
| Alarma de medicamentos | N^b^ | Y^c^ | Y | N | N | Y | Y | Y | Y | N | N | X^d^ | Y | N | Y | N | N | Y | N | N |
| Astursalud cita previa | N | Y | Y | N | N | Y | Y | Y | Y | N | N | X | Y | N | Y | N | N | Y | N | N |
| DiabetesPal | Y | N | N | Y | N | Y | N | Y | Y | X | X | Y | Y | X | X | X | X | Y | X | Y |
| FreeStyle librelink | Y | Y | Y | Y | N | Y | Y | Y | Y | N | Y | Y | Y | N | Y | Y | N | X | N | Y |
| Glucose Buddy Diabetes Tracker | Y | Y | Y | N | N | Y | Y | Y | Y | X | X | Y | Y | Y | X | X | X | Y | Y | Y |
| GVA +Salut | N | Y | Y | Y | N | Y | Y | Y | Y | N | Y | N | Y | N | Y | Y | N | X | N | Y |
| Medicamento Accesible Plus | Y | Y | Y | Y | Y | Y | Y | Y | Y | X | Y | Y | Y | Y | Y | Y | Y | Y | Y | Y |
| Medisafe | Y | Y | Y | Y | Y | Y | Y | Y | Y | X | Y | Y | Y | Y | Y | X | Y | X | Y | Y |
| Myfitness Pal | Y | N | Y | N | N | Y | N | Y | Y | Y | Y | Y | Y | Y | X | X | Y | X | X | Y |
| MyPills | Y | Y | Y | Y | Y | Y | Y | Y | Y | Y | X | Y | Y | X | Y | Y | X | Y | Y | Y |
| OneTouch Reveal | Y | Y | Y | X | Y | Y | Y | Y | Y | Y | Y | Y | Y | X | Y | Y | Y | Y | X | Y |
| Personalized Conversational Health Agent | N | Y | Y | Y | Y | Y | X | X | X | X | X | N | X | X | X | X | X | Y | X | X |
| SACYL Cita | X | N | X | N | N | Y | X | X | X | X | X | X | Y | X | X | X | X | X | X | X |
| Salud informa | Y | Y | X | Y | X | Y | Y | Y | Y | X | X | X | Y | X | Y | Y | X | X | X | Y |
| SCSalud | Y | Y | Y | Y | N | Y | N | X | Y | X | X | X | Y | X | Y | X | X | X | X | Y |
| SESCAM | Y | Y | Y | Y | N | Y | N | X | Y | X | X | X | Y | X | Y | X | X | X | X | Y |
| ScriptTalk | Y | Y | Y | Y | Y | Y | Y | Y | Y | Y | Y | Y | X | X | Y | Y | Y | Y | Y | Y |
|  |  |  |  |  |  |  |  |  |  |  |  |  |  |  |  |  |  |  |  |  |

| **Name of App** | **21** | **22** | **23** | **24** | **25** | **26** | **27** | **28** | **29** | **30** | **31** | **32** | **33** | **34** | **35** | **36** | **37** | **38** | **39** | **TOTAL YES** |
| --- | --- | --- | --- | --- | --- | --- | --- | --- | --- | --- | --- | --- | --- | --- | --- | --- | --- | --- | --- | --- |
| Alarma de medicamentos | N | N | X | X | X | N | N | Y | N | N | N | N | X | N | N | Y | Y | Y | N | **13** |
| Astursalud cita previa | N | N | X | Y | Y | N | N | Y | N | N | N | N | X | N | Y | Y | Y | Y | N | **16** |
| DiabetesPal | Y | X | Y | Y | Y | X | Y | Y | Y | Y | Y | Y | Y | Y | Y | N | X | Y | Y | **24** |
| FreeStyle librelink | Y | Y | Y | Y | Y | N | N | Y | Y | Y | Y | N | Y | N | Y | Y | Y | Y | N | **29** |
| Glucose Buddy Diabetes Tracker | Y | Y | Y | Y | Y | Y | Y | Y | Y | Y | Y | Y | Y | Y | Y | Y | X | N | X | **29** |
| GVA + Salut | N | N | Y | Y | Y | N | N | Y | Y | N | N | N | N | N | Y | Y | Y | Y | N | **22** |
| Medicamento Accesible Plus | Y | Y | Y | Y | Y | Y | X | X | X | Y | N | Y | Y | Y | Y | Y | X | Y | Y | **33** |
| Medisafe | Y | X | Y | Y | Y | X | X | Y | Y | Y | Y | Y | Y | Y | Y | Y | Y | Y | Y | **34** |
| Myfitness Pal | Y | Y | Y | Y | Y | X | X | Y | Y | Y | Y | Y | Y | Y | X | Y | Y | Y | X | **28** |
| MyPills | Y | X | X | Y | Y | X | X | Y | Y | X | Y | X | Y | Y | X | Y | X | Y | X | **27** |
| OneTouch Reveal | Y | Y | Y | Y | Y | X | X | Y | Y | Y | Y | Y | Y | X | Y | Y | X | Y | X | **31** |
| Personalized Conversational Health Agent | X | Y | X | X | X | N | N | Y | Y | Y | Y | Y | N | N | X | X | X | X | X | **12** |
| SACYL Cita | X | X | X | Y | Y | X | X | Y | X | X | X | Y | Y | X | X | X | X | Y | X | **8** |
| Salud informa | Y | X | X | X | Y | X | X | Y | Y | Y | Y | Y | Y | Y | Y | Y | Y | Y | X | **25** |
| SCSalud | Y | X | X | Y | X | X | X | Y | Y | X | X | X | Y | X | Y | Y | X | Y | X | **18** |
| SESCAM | Y | X | X | Y | X | X | X | Y | Y | X | X | X | Y | X | Y | Y | X | Y | X | **18** |
| ScriptTalk | Y | X | X | Y | Y | X | X | Y | Y | Y | Y | Y | X | Y | Y | Y | Y | Y | Y | **32** |

Note: ^a^These items are described in table 1; ^b^N= No; ^c^Y= Yes; ^d^X= Not applicable. The total score represents the number of MAG criteria met (“Yes” responses). Higher scores indicate better overall app quality across domains, whereas lower scores indicate more limited compliance.
